# Differences between parents’ and paediatricians’ perceptions of mild respiratory infections in childhood: contrast study

**DOI:** 10.3389/fpubh.2024.1377803

**Published:** 2024-05-09

**Authors:** Luis Ortiz-Gonzalez, Jesús Delgado-Ojeda, Mª Cinta Guisado-Rasco, Alicia Santamaria-Orleans, Cristobal Coronel-Rodríguez

**Affiliations:** ^1^Department of Biomedical Sciences, Medicine and Health Sciences Faculty, University of Extremadura, Badajoz, Spain; ^2^Medical Department, Laboratorios Ordesa S.L., Barcelona, Spain; ^3^Paediatrics Service, Amante Laffón Health Care Centre, Sevilla, Spain

**Keywords:** paediatric respiratory infections, family conciliation, paediatricians communication, respiratory infections prevention, paediatrics professional burden

## Abstract

**Introduction:**

Mild respiratory infections are a common reason for consultation in paediatrics, both in the emergency department and in primary care clinics. These conditions, mostly viral and self-limiting, have a significant impact on the healthcare system, school and work absenteeism, and family routines. Despite being common and banal illnesses from a medical perspective, they involve a significant concern in families. The main objective of the contrast study was to compare the perceptions of parents and paediatricians regarding mild respiratory infections in childhood and their impact on family conciliation.

**Materials and methods:**

Two online, cross-sectional surveys were conducted among Spanish paediatricians and parents with children aged 6 months to 12 years, involving 504 paediatricians and 1,447 families, with questions on attitudes towards visits to the paediatric consultation, care burden of minor pathologies, work, and family conciliation, and treatment and prevention of these illnesses.

**Results:**

Results showed significant differences in paediatricians’ and parents’ perceptions in many aspects. According to 34.5% of paediatricians and 27% of parents, families regularly go to the paediatrician without a scheduled visit. Only 4% of parents report having self-medicated their child, while paediatricians raise this percentage significantly to 48%. Regarding the question: “it is normal for a child to have an average of 4 colds a year,” only 25.5% of the surveyed families “strongly agree” unlike to 70.2% of paediatricians. 72.8% of paediatricians “strongly agree” with: “in my opinion, it is good for children to get sick to improve their immune system” reduced to 45.9% of parents. Consultations for minor pathologies represent a “high workload” for 60.9% of paediatricians, while this opinion is agreed by only 18.9% of the parents.

**Conclusion:**

Mild respiratory infections in childhood are perceived differently by paediatricians and parents. While paediatricians perceive them as a common and manageable phenomenon, parents tend to show higher concern and demand for medical attention. This study underlines the need to improve communication between paediatricians and parents to align perceptions, optimise the use of the health system resources, and improve the efficiency in the management of these common paediatric illnesses.

## 1 Introduction

Mild respiratory infections are one of the main reasons for consultation in the emergency departments and unscheduled paediatric primary care (1–5). Most of them are viral processes caused by respiratory syncytial virus, metapneumovirus, influenza, or adenovirus, among others, which resolve without requiring hospital care in immunocompetent children (6).

According to epidemiological studies, the paediatric population suffers an average of 4 to 8 mild infectious processes each year in the first 10 years of life, and even more during the first 3 years, especially if they are in school, attend nursery school or have siblings (1, 6, 7). Regarding mean duration of the processes and the estimated time to symptom resolution, this also varies depending on each child and pathology presented. Thus, in 90% of cases, a period of 2 to 7 days can be observed for the resolution of pharyngeal inflammation and up to 15–16 days for the resolution of a non-specific respiratory infection (8). This, along with the fact that many of these conditions are seasonally clustered, may give the impression that the child is always ill at certain times of the year and cause alarm to parents.

In addition to the impact on the health system (in terms of visits and prescriptions) and child’s health, those associated with school absenteeism are also relevant along with an occasional early return to school (7, 9, 10), as well as the absenteeism from work of their caregivers, and alterations in the routines of the family environment (parents, grandparents, and siblings) (11).

We are faced with a series of illnesses that are, most of them, common, recurrent, and banal from the paediatricians’ point of view, but which are a major cause for concern for families and, consequently, make them particularly demanding in terms of urgent medical care and prescriptions for the symptomatic treatment of this type of illness (antitussives, antipyretics, mucolytics, nasal decongestants, and antihistamines) or their prophylaxis using immune stimulants (12).

The aim of this study was to explore parents’ and paediatricians’ perceptions about mild respiratory illnesses in childhood, as well as to evaluate the impact of these attitudes and disparities on family conciliation.

## 2 Materials and methods

An online cross-sectional opinion survey was conducted simultaneously with Spanish paediatricians and parents with children between the ages of 6 months and 12 years during the last quarter of 2022.

To recruit physicians, a sample of more than 5,000 primary care (PC) paediatricians from all over Spain were invited to participate through social networks and mailings. Paediatricians responded to an online questionnaire about their daily clinical practise in the management of mild and common respiratory illnesses. Simultaneously to recruit parents, a second version of the same survey was launched through social networks and mailings to parents of children aged 6 months to 12 years and was complemented by sending the same questions to an online database of Spanish families with children in the same age range. This panel with ISO 26362, certifies the level of quality verified in methods of recruitment, panel organisation, treatment of the panellist, and the data. In the survey sent to the database of families, participants were recruited by invitation and on an as-needed basis to achieve representativeness. This approach avoids self-selection biases *per se*, as well as professional panellists and duplication. From the available respondent pool (100,000 panellists), a sample of families with children aged 6 months to 12 years was obtained that was representative by age, sex, area, habitat, and social class of the Spanish population. For subsequent analysis, this population was divided into three groups depending on the age of their children: 6 months to 2 years, 2–6 years, and 6–12 years. In the case of both paediatricians and parents’, all communications complied with current personal data protection regulations. Both online questionnaires (paediatritians’ and parents’) were hosted at Google Form platform and all the questions were compulsory.

To create the questionnaire, a search was carried out in the main databases: Pubmed, Embase, and the Cochrane Register of Controlled Trials (CENTRAL), and articles of interest were collected about mild respiratory conditions characteristic of childhood, impact on the paediatric care burden and family/work conciliation. The initial questionnaires had 20 questions, but the authors of the article, as coordinators, preferred to eliminate 3 of them since their content was very similar to other questions in the questionnaire and they were unified. The authors pre-tested the questionnaire among their acquaintances to validate it before launching it to the paediatricians’ and parents’ databases. The final questionnaires, consisted of 17 items, which were similar for both groups, except for minor changes/adaptations of the language to adjust them to each group. Questions covered attitudes of paediatricians and parents regarding visits to the physician, care burden of mild respiratory illnesses, work and family conciliation concerning respiratory illnesses, impact on school attendance, treatment expectations, self-medication, and criteria of normality concerning recurrent mild respiratory illnesses in childhood. Table 1 shows the different statements of the items in the paediatricians’ and parents” survey. The contents of questionnaires were processed and analysed together. To adapt the questions to each target, one question was not exactly the same in both questionnaires, making not possible a direct comparison of the data (see Table 2). One question had different response possibilities. In the family survey, the following 2 responses were possible: “No” and “Yes, sometimes.” However, the paediatrician survey provided 5 possible responses: “No, never,” “Hardly ever,” “Sometimes”; “Regularly” and “Very often.” To compare the answers, had to be homogenized, and for that purpose, in the paediatricians’ response, “Hardly ever,” “Sometimes,” “Regularly” and “Very often” were considered equivalent to the “Yes, sometimes” response of the family survey (see Table 3). To compare the questions, a chi-square goodness-of-fit test was performed, comparing the data collected from the parents with the data collected from the paediatricians, taking the latter as the population reference. As a result of this comparison, a *p*-value <0.0001 was obtained, confirming that the information reported by the parents vs. that of the paediatricians consulted was significantly different.

**Table 1:** Study questionnaires.

Paediatricians	Parents
Of all patients ≤12 years old consulting for mild/moderate respiratory conditions, please indicate the % of them attending WITHOUT a scheduled visit (as an emergency)	Normally, how do you go to the paediatrician when your child gets sick?
I believe that parents misuse emergency departments or unscheduled visits for minor illnesses	I believe that families misuse emergency departments or unscheduled visits for minor illnesses
Consultations for minor illnesses place a heavy workload on paediatricians	Consultations for minor illnesses place a heavy workload on paediatricians
Most of the consultations for minor illnesses can be solved remotely (by email or telephone)	Paediatricians resolves most of the consultations for minor illnesses remotely (by email or telephone)
If the patient has to be absent from school, do you usually discuss with the parents how to reconcile work and family life?	If your child is unable to go to school, do you often talk to your paediatrician about how you reconcile work and family life at home?
Parents should plan how to organise themselves when a child is unwell and cannot go to school	Parents should plan how to organise themselves when a child is unwell and cannot go to school
I am aware of the disruption that minor illnesses can cause in the family environment	My paediatrician is aware of the disruption that minor illnesses can cause in my family environment
Has any family ever admitted to you that they have self-medicated their children and taken him/her to school even though they were not completely well?	Have you ever self-medicated your child and taken him/her to school even though he/she was not well?
Do you think that sometimes children return to school/daycare center even if they have not fully recovered from their mild/moderate respiratory conditions?	Has your child ever returned to school/daycare center despite not having fully recovered?
I recommend that parents send their children to school as late as possible	My paediatrician recommends that I send my children to school as late as possible
It is normal for a child to have an average of 4 colds per year	It is normal for a child to have an average of 4 colds per year
In my opinion, it is good for children to get illnesses in order to improve their immune system	In my opinion, it is good for children to get illnesses in order to improve their immune system/defences
It worries me when a child has 4 or more colds a year	It worries me when my child has 4 or more colds a year
Families are well aware that young children can get one cold after another and should not worry about it	Families are well aware that young children can get one cold after another and should not worry about it
Of all patients ≤12 years old consulting for mild/moderate respiratory conditions, what percentage of them do you usually prescribe treatment for?	When you go to the paediatrician for a minor health problem, what percentage of the times is your child prescribed any treatment?
In your experience, do the parents of your patients <12 years old, who present for minor respiratory complaints (e.g., common cold, cough and mucus, etc.) for which you do not consider treatment necessary, usually ask you to prescribe something to their children?	If your paediatrician does not prescribe any treatment for your child, do you usually ask him/her to prescribe something?
What type of treatment do they most commonly request?	In these cases, what kind of treatment do you usually ask for?

Paediatricians’ version and parents’ version.

**Table 2:** Responses to the questionnaires by paediatricians and parents on their attitudes regarding visits to the physician (this question was not the same in both questionnaires).

Paediatricians questionnaire	Parents questionnaire
Of all patients ≤ 12 years old consulting for mild/moderate respiratory conditions please indicate the % of them attending WITHOUT a scheduled visit (as an emergency).	How do you usually go to the paediatrician when your child gets sick?
34.5% (average value)	72.8%—I usually go WITH scheduled visit 27.2%—I usually go WITHOUT a scheduled visit
*p <*0.0001	

Data expressed as percentage. METHODOLOGICAL DISCLAIMER: Given that the question was not exactly the same in both questionnaires, it was not possible to make a direct comparison of the data. In order to compare these two items, a chi-square goodness-of-fit test was performed, comparing the data collected from the families with the data collected from the paediatricians, taking the latter as the population reference. As a result of this comparison, a *p* < 0.0001 was obtained, confirming that the information reported by the families vs. that of the paediatricians consulted was significantly different.

**Table 3:** Responses to the questionnaire by paediatricians and parents about treatments for mild respiratory infections.

Of all patients < 12 years old consulting for mild/moderate respiratory conditions, what percentage of them do you usually prescribe treatment for?		
Paediatricians	34.7 (27.6)	*p* < 0.0001
Parents	46.5 (31.4)	

Do the parents of patients < 12 years old who present for minor respiratory complaints, for which you do not consider treatment necessary, usually ask you to prescribe them something?*			
	No	Yes	
Paediatricians	0.6	99.4	*p* < 0.0001
Parents	78.2	21.8	

What type of treatment do they most commonly request?			
	Etiological	Symptomatic	Preventive
Paediatricians	37.3	76.4	45.9
Parents	8.6	86.7	9.2
Significance	*p* < 0.0001	*p* < 0.00021	*p* < 0.0001

Data expressed as percentage and mean (standard deviation). *METHODOLOGICAL CLARIFICATION: This question had different possible answers for parents (“No” and “Yes, sometimes) and for paediatricians (“No, never,” “Hardly ever,” “Sometimes”; “Regularly” and “Very often.”). To make the comparison, answers were homogenized, and paediatricians’ response “Hardly ever,” “Sometimes”; “Regularly” and “Very often” were considered equivalent to the parents’ response “Yes, sometimes.”

Categorical variables were reported as frequencies and percentages. Variables with a normal distribution were expressed as mean ± standard. A comparison between the variables was conducted using Fisher’s exact test for categorical variables, the Student’s *t*-test, or the ANOVA test for continuous variables, depending on whether the comparison involved two or more groups, respectively. All statistical analyses were performed using the SAS statistical package version 9.4, with a statistical significance value set at 0.05.

## 3 Results

A total of 504 paediatricians participated with a mean age of 51.4 ± 11 years, of which 84.5% of them reported having children and most had children over the age of 6 years (89.4%). About 64.1% worked in the public sector, 22% in the private sector and 13.9% in both. A total of 1,447 families also participated, with a mean age of the parent who answered the questionnaire of 39.3 ± 6 years, of which 72.9% lived in an urban environment and 60.1% had a university education. It should be noted that 88.6% of the sample were two-parent families and 72.8% of the families lived with or near relatives. Regarding their employment status, 71.2% were in paid employment, 21% were not working and 7.8% were self-employed. 61.7% were not teleworking at the time of answering the survey. Tables 4, 5 shows the profiles of the participating paediatricians and parents.

**Table 4:** Profile of participating paediatricians.

Participating paediatricians	504		
Age	51.37 (19.96) years		
Have children	84.5%		
Age of children	<2 years	3–5 years	≥ 6 years
	13.4%	17.4%	89.4%
Area of work	Public	Private	Both
	64.1%	22%	13.9%

Data expressed as percentage and mean (standard deviation).

**Table 5:** Profile of participating parents.

Participating parents	*N* = 1,447		
Respondent’s age	39.33 (6.03) years		
Respondent’s sex	Male	Female	
	16.8%	83.2%	
Age of children	< 2 years	3–5 years	≥ 6 years
	50.4%	35%	52.5%
Environment/habitat*	Rural area	Semi-urban area	Urban area
	6.7%	20.4%	72.9%
Educational level	Primary education	Secondary education	University/higher education
	12.4%	27.5%	60.1%
Employment status	Active/self-employed	Not active/unpaid domestic work	Active/employed
	7.8%	21%	71.2%
Teleworking time	None	Partial	Full time
	61.7%	32.4%	6%
Type of family	Single-parent	Two-parent	
	11.4%	88.6%	
Living with relatives	Living with relatives	Do not live with or have relatives nearby	Have relatives nearby
	8.2%	27.1%	64.8%

Data expressed as percentage and mean (standard deviation). *Rural area (municipality of < 2,000 inhabitants); Semi-urban area (municipality with 2,000–10,000 inhabitants); Urban area (municipality with more than 10,000 inhabitants).

### 3.1 Attitudes of paediatricians and parents regarding visits to the physician

Regarding the attitude of parents and paediatricians to visits to health centres (Tables 2, 6), 27.2% of the parents considered that they regularly visit the paediatrician for mild respiratory conditions without a scheduled visit (emergency visits); for paediatricians, this percentage reached 34.5%. Most of the paediatricians (72.8%) strongly agreed that families misuse emergency or walk-in visits for minor illnesses compared to only 22.9% of the parents (*p* < 0.0001). Likewise, 60.9% of the paediatricians vs. 18.9% of the parents strongly agreed or agreed that consultations for minor illnesses place a heavy workload on paediatricians (*p* < 0.0001). Paediatricians’ assessment of this last point varied depending on whether the paediatricians had children and their ages. The group who strongly agreed or agreed represented 88.8% for paediatricians with children ≤12 years, 87.7% for paediatricians with children >12 years, and 93.4% in the group without children (*p* = 0.006). This data also varied depending on the place they worked (93.5% for public health centres vs. 74.78% for private ones, *p* < 0.0001).

**Table 6:** Responses to the questionnaire by paediatricians and parents on their attitudes regarding visits to the physician.

	Strongly agree	Agree	Neither agree nor disagree	Disagree	Strongly disagree	
I believe that parents misuse emergency departments or unscheduled visits for minor illnesses						
Paediatricians	72.8	23.4	2.2	1	0.6	*p* < 0.0001
Parents	22.9	28.9	20.6	19.6	8	
Consultations for minor illnesses place a heavy workload on paediatricians						
Paediatricians	60.9	28	6.3	3.4	1.4	*p* < 0.0001
Parents	18.9	31.4	25.3	16	8.4	
Most minor consultations can be solved remotely (by email or telephone)						
Paediatricians	18.1	38.3	20.6	17.5	5.5	*p* < 0.0001
Parents	14.7	30.3	22.7	19.4	12.9	
It is normal for a child to have an average of 4 colds per year						
Paediatricians	70.2	22.2	2.4	3.8	1.4	*p* < 0.0001
Parents	25.5	43.8	14.1	13.6	3	
In my opinion, it is good for children to get illnesses in order to improve their immune system						
Paediatricians	12.5	26.2	27.4	23	10.9	*p* < 0.0001
Parents	11.8	34.1	29.6	16.7	7.8	
It worries me when a child has 4 or more colds a year						
Paediatricians	7.7	18.7	20.1	25.8	27.6	*p* < 0.0001
Parents	30.5	28.4	22.2	12.6	6.3	
Parents are well aware that young children can get one cold after another and should not worry about it						
Paediatricians	13.9	26.2	21.8	28.4	9.7	*p* < 0.20755
Parents	12.1	29.9	26.7	21.8	9.5	

Data expressed as percentage.

### 3.2 Care burden of mild respiratory illnesses

In terms of burden of care, 56.4% of the paediatricians vs. 45% of the parents agreed or strongly agreed that most consultations for minor illnesses could be resolved remotely (by email or telephone). Likewise, 5.5% of paediatricians vs. 12.9% of parents strongly disagreed with this statement (*p* < 0.0001).

### 3.3 Criteria of normality concerning recurrent mild respiratory illnesses in childhood

In addition, when asked about what was considered as “normal” in children’s infections prevalence, different views were expressed by paediatricians and parents, in this context, 38.2% of paediatricians recommended families send their children to school as late as possible, in contrast to 17% of parents who considered that this was the recommendation they received in the paediatric consultation (*p* < 0.0001). Secondly, 38.7% of the paediatricians vs. 45.9% of the parents strongly agreed or agreed that it is good for children to get illnesses to strengthen their immune system (*p* < 0.001) and 25.5% of the parents vs. 70.2% of the paediatricians considered normal for a child to have an average of 4 colds per year (*p* < 0.0001). In line with this figure, 30.5% of parents were very concerned that their children have 4 or more colds per year. However, this concern is only shared by 7.7% of paediatricians, most of whom are not worried about a child having 4 or more colds a year (*p* < 0.0001). Likewise, 40% of the parents and paediatricians agreed that young children can get one cold after another and that it is not relevant (strongly agree or agree) and, conversely, 38% of the paediatricians and 31.3% of the parents disagreed about downplaying its importance.

### 3.4 Work and family conciliation concerning respiratory illnesses

We found discrepancies on how mild respiratory infections affect work-family conciliation (Table 7). It is relevant that 83.8% of the parents vs. 23.9% of the paediatricians indicated that they never or hardly ever talk in the paediatric consultation about reconciling work and family life if a child is unable to go to school. Only in a small percentage of cases (6.4%) parents recognised that they regularly discuss work-life conciliation with their paediatricians. By contrast, paediatricians’ view was that they are more concerned about work-life conciliation, and 35.4% of paediatricians considered that they usually discuss these issues with their families (*p* < 0.0001). Regarding the need for having a plan for the moments when the children are ill and must stay at home, 83.3% of paediatricians agreed with this question and considered that parents should plan how to organise themselves when a child is unwell and cannot go to school. The percentage of parents who agreed with this statement dropped to 58.7% (*p* < 0.0001).

**Table 7:** Responses to the questionnaire by paediatricians and parents on work/family conciliation.

	Very often	Regularly	Sometimes	Hardly ever	No, never	
If the patient has to be absent from school, do you usually discuss with the patient’s parents about how to reconcile work and family life?						
Paediatricians	6.6	28.8	40.9	17.5	6.4	*p* < 0.0001
Parents	1.7	4.7	9.8	15.3	68.5	
My paediatrician is aware of the disruption that minor illnesses can cause in the family environment						
Paediatricians	58.3	34.1	4.6	1.6	1.4	*p* < 0.0001
Parents	10.8	19.2	30.9	15.8	23.3	
Has any family ever admitted to you that they have self-medicated the child and taken him/her to school even though the child was not well?						
Paediatricians	16.3	44.3	35.6	2.6	1.2	*p* < 0.0001
Parents	0.7	3.5	26.3	21.9	47.6	
Do you think that sometimes children return to school/daycare even if they have not fully recovered from their mild/moderate respiratory conditions?						
Paediatricians	27	55.3	14.9	2.4	0.4	*p* < 0.0001
Parents	1.7	7.7	27.9	24.7	38	
I recommend that parents send their children to school as late as possible						
Paediatricians	17.5	20.7	25.4	19.3	17.1	*p* < 0.0001
Parents	7.2	9.8	34.7	14.8	33.5	
Parents should plan how to organise themselves when a child is unwell and cannot go to school						
Paediatricians	50	33.3	10.9	3.8	2	*p* < 0.0001
Parents	25.8	32.9	15.9	14.3	11.1	

Data expressed as percentage.

While 94.4% of paediatricians considered that they were aware of the disruption that minor illnesses can cause in the family environment (agree or strongly agree), only 30% of parents had the same perception. Almost 4 in 10 parents (39.1%) vs. 3% of paediatricians considered that paediatricians are not aware of the disruption that minor illnesses can cause in family environment (*p* < 0.0001).

### 3.5 Self-medication and impact on school attendance

According to the parents, only 4.2% of them had regularly/very regularly self-medicated their children and taken him/her to school despite they were not completely well. However, according to paediatricians, 60.6% of parents had acknowledged that they do this on a regular/very regular basis (*p* < 0.0001). Percentage of paediatricians to whom parents had acknowledged that they very often take their children to school despite they are not completely recovered is higher among paediatricians who do not have children (21.1%), compared to paediatricians who have children (16.1%) (*p* = 0.03691).

It should also be noted that almost one in two parents (47.6%) stated that they had never self-medicated their children and taken him/her to school despite not being completely recovered while for paediatricians this profile was less than 1.2% (*p* < 0.0001).

Parents in active employment (employed or self-employed) are the ones who most frequently reported having taken their children to school when not fully recovered on some occasion, compared to non-active households (unemployed, on sick leave, retired and/or domestic worker) in a percentage of 32.4% for employed, 31.8% self-employed and 23.4% for non-active households, respectively (*p* < 0.005). Similarly, families made up of several family members, who have relatives close did it in a lower frequency versus those who neither live together nor have relatives close by, with a percentage of 25.4, 25.8, and 36%, respectively, (*p* < 0.082).

Likewise, 9.4% of parents recognised that their children regularly or very often return to school/daycare centres despite not being fully recovered. The frequency with which parents took children to school without being fully recovered decreased with children’s age in a statistically significant way (*p* = 0.03240). In contrast, 82.3% of paediatricians considered that children return to school/daycare centres regularly or very regularly, even if they have not fully recovered from their mild/moderate respiratory conditions (*p* < 0.0001).

Those families in which parents worked outside the home were asked how they usually organise themselves in case of sudden illness of their children (Table 8). In this case, 43% of the participants indicated that the grandparents or relatives took care of the children while they were ill, 34.6% that the mother/father took time off work to take care of the child, 18.9% that the father/mother teleworked and could therefore stay with the child, and external help (e.g., babysitting) was only used to take care of the child in 3.4% of the cases. These percentages varied depending on the age of the children, with parents being those who cared for them more often when they were younger.

**Table 8:** Responses of parents to the question on how they organise themselves in case of sudden illness of their child when they work outside the home based on the age of the children.

If both parents work outside the home, how do you usually organise yourselves if your child suddenly becomes ill? (%)					
	< 6 months	6 months–2 years	3–5 years	6–12 years	≥ 13 years
Mother/father takes time off work to take care of the child	25	33.9	35.1	35.7	30
Grandparents or relatives care for the child while he/she is ill	25	46.2	41.1	39.4	30
External help (e.g., babysitter) to take care of the child	0	3.5	3.4	2.5	30
Parent teleworks so they can stay with the child	50	16.4	20.3	22.3	10
*p* = 0.0166					

Data expressed as percentage.

### 3.6 Treatment expectations

According to the paediatricians, on average, they prescribe some treatment to 34.7% of all patients ≤12 years of age who consult for mild/moderate respiratory conditions (Table 3). This percentage was lower for paediatricians working in public centres (29.7%) than for the ones working in private clinics (45.3%) (*p* < 0.0001). In comparison, 46.5% of the surveyed parents pointed out that their paediatricians prescribe some treatment when they visit him/her regarding a minor health problem in their child (*p* < 0.0001). On the word of the paediatricians, only 5% of parents never or hardly ever ask them to prescribe a treatment (for a minor respiratory condition such as a common cold, cough, or mucus) when they have considered it unnecessary, with significant differences in this question depending on whether the paediatricians have children or not, and the ages of their children. Likewise, 77.6% of the paediatricians without children considered that parents regularly or very often ask them for a prescription if they have not considered it, vs. 68.7% of paediatricians with children <12 years old and 60.9% for paediatricians with children ≥12 years old (*p* = 0.00053). By contrast, 78.2% of the parents considered that they respect the paediatrician’s decision of not prescribing any treatment for their children and do not ask him/her to prescribe something for them (*p* < 0.0001). If some type of treatment is requested, 37.3% of the parents request an aetiological treatment and only 8.6% of the parents admitted that they request a curative treatment such as an antibiotic if the paediatrician does not consider it necessary to prescribe any treatment. As regards preventive treatments, to reinforce defenses, according to the paediatricians, 45.9% of parents request this type of treatment, in contrast to the 9.2% of parents who believe it (*p* < 0.0001).

Request for treatment by families depends on the age of their children. As the age of the children grows (from 6 months to 12 years), the incidence of requesting treatment to alleviate symptoms, such as mucolytics and antitussives, increases, in a statistical way (84.9% for parents with children between 6 months and 2 years, 88.9% for parents with children between 3 and 5 years and 91.2% for parents with children between 6 and < 12 years, *p* = 0.01380).

## 4 Discussion

Mild respiratory illnesses in childhood are a major healthcare burden for paediatricians (2, 13) even though they are not perceived as such by families. Approximately 20% of children develop symptoms compatible with viral infections (respiratory syncytial virus, influenza, adenovirus, or metapneumovirus) that generate demand for emergency care or unscheduled visits to the paediatrician and, on occasion, hospitalization (14–16). Perception of the use of unscheduled visits (emergencies) by parents is much lower than that perceived by paediatricians—among whom statistically significant differences were found depending on whether they work in private practises or public centres (87.3% vs. 99.1% respectively, *p* < 0.0001)—who consider that families misuse emergency departments or unscheduled visits for minor illnesses. Excess of paediatric emergencies going to hospitals has been the subject of numerous studies and inefficiency of paediatric primary care system in carrying out this filter has been proposed as the cause of this situation (2). In our setting, in addition to private paediatric care, we find significant variability in the availability of care at the public centres, so the paediatrician’s perception may be influenced by the work environment (health centres with paediatric teams with 7-h working days, others with 2-h working days, with morning care, until 5 p.m., until 9 p.m. or those providing continuous 24-h emergency care) (2). The pressure on health care services due to emergency consultations at health centres (or “walk-in consultations”) varies from 3 to 25%, of which only 44.3% would have a justified reason to be “urgent” or undelayable (2). The active role of families in going directly to the paediatric emergency department for non-urgent conditions may also play a role in this over-demand (2). The unpredictability of the appearance of any medical condition is indisputable, but it is essential to raise awareness among parents about the optimal use of healthcare resources and minimise, as far as possible, the use of the emergency department.

An increasingly common and interesting alternative in mild respiratory illnesses in childhood is the use of non-face-to-face diagnostic and treatment tools. The recent COVID pandemic forced the implementation of telemedicine systems and demonstrated their effectiveness in caring for a large number of patients (17). Despite significant differences, a large percentage of the two surveyed collectives considered that most consultations for minor ailments could be resolved remotely (by email or telephone). It is interesting to encourage this type of solution to provide quick responses to families and help to reduce the burden of care for paediatrics professionals.

A child’s recurring health concerns often have a major impact on the whole family. In addition to the child’s own discomfort, there is the possibility of a social, emotional, and financial impact on family members (4, 18, 19) which, although not as significant as that of parents of children with chronic conditions, can cause families to sometimes feel overwhelmed and exhausted by sleep disturbances as they try to manage their day-to-day work and family life (18, 19). Furthermore, many parents are concerned about lost school time and the disruption to work caused by these disorders (4, 18–20).

Therefore, in the present study, it was noteworthy that the two analysed groups had quite different opinions on aspects related to work-life conciliation. Almost 70% of parents reported that they never discuss work-life conciliation with their paediatricians, even though 94.4% of them consider themselves aware of the disruption that minor illnesses can cause in the family setting. Despite some articles refer to the role of parents and paediatricians in children’s care and how the latter can involve more parents in the overall care of the patient, they do not refer to how to approach family work-life conciliation (21, 22). It is important to work on different tools that improve communication between paediatricians and parents, to reduce this gap and bring their realities closer together.

As a result of the difficulties in achieving a work-life balance, it is understandable that parents may resort on occasion to self-medicating their children, even accelerating their return to school or nursery without them having fully recovered and without waiting for the contagious period of the illness to pass. Undoubtedly, premature return of the children to childcare facilities should be avoided in order to prevent the transmission of the infectious agent to the community, as well as for the children’s wellbeing (9, 10). However, the lack of self-perception of this reality on the part of parents is striking, as only 4.2% of them recognised this practise on a regular or very regular basis, and almost half of them (47.5%) considering that they had never done it. Several studies place self-medication in ranges from 7.1 to 58.8% (23–26) in line with the perception of paediatricians, who considered that more than 60% of parents regularly self-medicate their children. Information needs to be provided to make parents aware of the need to resort to self-medication only on very specific occasions and the importance of waiting until children are fully recovered before returning them to school (9, 10).

According to the data collected in this study, there was a great interest on the part of parents in obtaining treatment for their children’s disorders through the paediatrician, in spite of it was not initially prescribed. Paediatricians reported that practically all parents ask for treatment, while only one in five parents acknowledged this in our study.

Regarding the type of treatment requested, paediatricians and parents agreed that symptomatic treatment was the one that most (8 out of 10). As the age of the children grew, the request for treatment to alleviate symptoms, such as mucolytics and cough suppressants, also increased. This type of treatment is also the most used in other countries, such as Germany (32.1%) (25) or Australia (40%) (27).

Despite campaigns for the rational use of antibiotics, paediatricians reported that parents continue to request prescriptions for them, although they did not recognise it as often (37.3% vs. 8.6%). Fortunately, real use of self-medicated antibiotics in European countries is about 8% (28), which is interestingly in line with the percentage of parents who mentioned actively requesting them in our study, and much lower than in other countries (34% in Eastern European countries, 22% in Africa, or 17% in South America) (28, 29).

Finally, paediatricians reported high demand for treatments that strengthen the immune system and according to their experience, one in two parents ask for preventive treatment. The percentage of parents who admitted to requesting it as such is much lower, although interestingly it increases with the age of the children (from 9.4% in children under 2 years old to 13.8% in children aged 6 to 12 years old). The experience accumulated by parents of multiple infectious illnesses over the years may make them interested in requesting preventive treatment rather than symptomatic one. In our setting, 87.4% of paediatricians actively recommended the use of immune stimulants, with 21.5% of these recommendations being at the request of the parents (12).

An interesting section is the one that refers to the perception of normality in children’s infections. Children are born with an immature immune system and will consequently suffer from a quite high number of infections during the first months/years of life. Healthy children without underlying disease have an average of 6–8 upper respiratory tract infections per year, which can reach 10–12 infections if the child attends nursery school, has younger siblings, or has a predisposing factor such as asthma, which can sometimes give the impression that the child is always ill and make parents question the normality of the case (6, 7). The majority of these recurrent processes in immunocompetent children are usually viral, banal, transient, and self-limiting (6, 7, 30). However, it should be remembered that, in the presence of certain risk factors such as the existence of a relevant family history and/or consanguinity, multiple organ involvement, affectation of the weight curve, or the lack of clear improvement between episodes, there may be suspicion of a significant underlying immunological defect and such episodes should not be trivialised (6, 7, 30).

In this regard, for one in four surveyed parents, it was totally normal for children to have an average of 4 colds a year and almost half of them even consider that it is good for children to get illnesses as a way of strengthening their immune system. Perceptions of paediatricians slightly and significantly differed, giving more normality to the fact that children have an average of 4 colds, but downplaying the belief that it is good for them to get illnesses to improve their immune response. The positive or negative effect of frequent infections in early life is controversial, because although with normal maturation of the immune system on reaching school age the rate of infections in children should not differ from that in adults (6, 7), it has been observed that early exposure to bacteria, viruses and other organisms helps to strengthen the immune response to allergens or proteins that trigger allergic reactions (the “hygiene” theory). However, early contact with these substances and subsequent infection can affect lung function and make the child more susceptible to asthma later in life (31–33). For this reason, it could be helpful for paediatricians to inform parents about the usual periodicity of respiratory infections in the first months/years of life to help them normalise and contextualize the repeated mild infectious processes common in this age group.

Regarding the representativity of our results, a possible limitation of the study is that it is based on voluntary responses from the surveyed Spanish paediatricians and parents that may not reflect the general point of view, although the large number of responses minimises such bias.

## 5 Conclusion

Respiratory infections in childhood are a common phenomenon whose dimensions were perceived differently between paediatricians and parents in most of the questions asked in this study. The tendency for parents to continue requesting treatments for these illnesses even if they are not really necessary stands out. Symptomatic treatments and those to stimulate the immune system are the most frequently requested by parents. It is relevant to improve communication between paediatricians and parents to bring together positions regarding the normal prevalence of mild respiratory infections during childhood, the burden of care they place on the healthcare system, the family-work conciliation, and especially on the risks of self-medication in children, thereby encouraging the rational use of medicines.

## Data availability statement

The original contributions presented in the study are included in the article/supplementary material, further inquiries can be directed to the corresponding author.

## Ethics statement

Ethical approval for the studies involving humans was not required because parents and paediatricians consent was waived due to the fact that all of the families and paediatricians accepted the offer to participate in this survey the moment they registered in the database used for this study. The studies were conducted in accordance with the local legislation and institutional requirements.

## Author contributions

LO-G: Conceptualization, Data curation, Formal analysis, Methodology, Writing – original draft, Writing – review & editing. JD-O: Validation, Writing – review & editing. MCG-R: Methodology, Writing – review & editing. AS-O: Conceptualization, Project administration, Writing – original draft, Writing – review & editing. CC-R: Supervision, Validation, Writing – review & editing.
